# Modification in the Motor Skills of Seniors in Care Homes Using Serious Games and the Impact of COVID-19: Field Study

**DOI:** 10.2196/36768

**Published:** 2022-05-10

**Authors:** Jana Marina Kleschnitzki, Inga Grossmann, Reinhard Beyer, Luzi Beyer

**Affiliations:** 1 Institute of Psychology Faculty of Human Sciences Humboldt-University of Berlin Berlin Germany; 2 Department of Science All About Me GmbH Berlin Germany; 3 Department of Quantitative Research Methods Alice-Salomon University of Berlin Berlin Germany

**Keywords:** serious games, motor skills, motor, long-term care, exercise, movement, coronavirus effects, eHealth, seniors, older adult, elder, senior population, aged, care home, intervention effects analysis, COVID-19, pandemic, digital game, digital health, physical activity

## Abstract

**Background:**

The pandemic has highlighted the importance of low-threshold opportunities for exercise and physical activity. At the beginning of 2020, the COVID-19 pandemic led to many restrictions, which affected seniors in care facilities in the form of severe isolation. The isolation led, among other things, to a lack of exercise, which has led to a multitude of negative effects for this target group. Serious games can potentially help by being used anywhere at any time to strengthen skills with few resources.

**Objective:**

The aim of this study is to evaluate the effectiveness of a serious game to strengthen motor skills (study 1) and the influence of pandemic restrictions (study 2) on seniors in care facilities.

**Methods:**

The data on motor skills (measured by the Tinetti test) originated from an intervention study with repeated measurements that was interrupted by the pandemic conditions. Data were collected 4 times every 3 months with an intervention group (IG, training 3 times for 1 hour per week) and a control group (CG, no intervention). There were 2 substudies. The first considered the first 6 months until the pandemic restrictions, while the second considered the influence of the restrictions on motor skills.

**Results:**

The sample size was 70. The IG comprised 31 (44%) participants, with 22 (71%) female and 9 (29%) male seniors with an average age of 85 years. The CG comprised 39 (56%) participants, with 31 (79%) female and 8 (21%) male seniors with an average age of 87 years. In study 1, mixed-design ANOVA showed no significant interaction between measurement times and group membership for the first measurements (*F*_2.136_=1.414, *P*<.25, partial η2=.044), but there was a significant difference between the CG (mean 16.23, SD 1.1) and the IG (mean 19.81, SD 1.2) at the third time of measurement (*P*=.02). In study 2 the mixed-design ANOVA (used to investigate motor skills before and after the pandemic conditions between the 2 groups) couldn’t reveal any significant interaction between measurement times and group membership: *F*_1.67_=2.997, *P*<.09, partial η2=.043. However, there was a significant main effect of the time of measurement: *F*_1.67_=5.44, *P*<.02, partial η²=.075.

**Conclusions:**

During the first 6 months, the IG showed increased motor skills, whereas the motor skills of the CG slightly deteriorated and showed a statistically significant difference after 6 months. The pandemic restrictions leveled the difference and showed a significant negative effect on motor skills over 3 months. As our results show, digital games have the potential to break down access barriers and promote necessary maintenance for important skills. The pandemic has highlighted the importance of low-threshold opportunities for exercise and physical activity. This potentially great benefit for the challenges of tomorrow shows the relevance of the topic and demonstrates the urgent need for action and research.

**Trial Registration:**

Deutsches Register klinischer Studien DRKS00016633; https://tinyurl.com/yckmj4px

## Introduction

### Background

By the spring of 2020, the rapid spread of coronavirus led several German states to implement drastic measures in order to protect their population. Many of these measures included restrictions on movement and social contact, which particularly impacted groups that were already affected by multimorbidity, restricted functionality, and the need for care [1]. For instance, the high mortality rate, in particular among the older, more vulnerable population (6.5% of the population 65+ years old and 14% of those 75+ years old) [2,3], put a considerable burden on the health care system. This tragically resulted in an increase in restrictions stipulated and implemented by local authorities. Especially in nursing homes, caution and strict adherence to regulations were essential [4].

### Physical Consequences of the Pandemic-Related Isolation

Compared to the results from a survey study conducted in 2017, the pandemic-related measures led to a sharp reduction in physical activity [5-7] in 40% of individuals between the ages of 46 and 90 years, a 29% increase in sitting time, and a widespread reduction in leisure activities [8-11]. By now, a number of negative effects of the measures implemented during the pandemic are known and documented. The literature, for instance, provides evidence of an exacerbation of psychological issues with possibly persistent, long-term effects [12-14], which are also associated with increased depressive and anxiety symptoms [15-19]. At the same time, due to physical inactivity, more negative physical symptoms have been found [20,21]. These, together with other mentioned effects, lead to increased mortality [22] and reduced quality of life [23], which can be additional triggers for withdrawal and physical inactivity (a vicious circle) [4,24]. An effective strategy to counteract these harmful effects is to keep up regular physical activity. It can promote resilience (self-efficacy and optimism) and mental health [25-27], reduce depressive symptoms [28], and balance an individual’s overall psychomotor performance [1]. During the pandemic [29], even light physical activity can mitigate the negative effects [8]. Both the global recommendations for physical activities published by the World Health Organization (WHO) and the German National Health survey (*Bundes-Gesundheitssurvey*) issued by the Robert Koch Institute [30] point out that physical activity is key for seniors to maintain not only their health and mobility but also their independence and self-reliance [30,31], especially in times of a pandemic [32]. To be sustainable, the latter should be integrated into daily routines and carried out collectively [30].

### Social Consequences of the Pandemic

Even before the outbreak of the COVID-19 pandemic, the German health care system was faced with major challenges related to demographic change. Among those were the growing number of aging individuals in need of care (22% of 60-80-year-olds) [33] and, as a result, the increased utilization of health care services, which burdened the social security system and the health care structure [34]. Moreover, the rapidly growing need for support caused by the pandemic has placed excessive demands [35] on practitioners, creating an enormous amount of stress, which has negatively impacted the quality of care and, by extension, also the psychological well-being of seniors [36]. The forecasted shortage of half a million skilled workers in the German nursing sector by 2035 is also alarming, given that it may further exacerbate the issue [37]. Therefore, it is imperative to focus on health care promotion for seniors, which concentrate on health care resources that promise long-term autonomy and independence [38].

### Effectiveness of Digital Serious Games That Promote Physical Activity

The positive effect of physical activities [39] combined with the challenges outlined before highlight the need for low-threshold, inexpensive, nationwide solutions that promote physical activity [40]. Digital technologies can play a key role in this effort [41]. These new digital technologies in the health care sector that primarily focus on prevention and health promotion via gameful experiences can be summarized under the umbrella term “serious games for health” [42].

The overall positive effect of serious games with a special focus on promoting motor movement sequences in older individuals has already been investigated and reported in several meta-analyses [43-47] that highlighted that the impact resulting from the use of serious games can range from moderate-to-strong training effects, especially in participants with initially low motor capability [48]. Vaziri et al [49] specifically point to a significant reduction in the risk of falling, especially in participants with low initial motor capability [50], while Stanmore [50] and Brox [51] were even able to report the first positive follow-ups in this regard. However, it needs to be emphasized that long-term effectiveness of physical activity has not been sufficiently investigated yet [52-54].

Initial studies conducted during the pandemic show the usefulness and positive effects of digital exercise on physical well-being [55]. For example, Parker et al [56] reported that participants who used digital exercise platforms were more likely to remain physically active during the pandemic. Thus, serious games can be part of a rehabilitative treatment plan that might prevent the functional deterioration of seniors suffering from COVID-19 [57]. In conclusion, it can be said that most of the findings indicate promising potential for using game-based interventions with senior citizens, thus highlighting the importance of establishing such opportunities, especially during the pandemic [58]. However, there is a clear need for research into the long-term effects [59], the study conditions [45], and the intensity and choice of suitable game modules relative to different physical conditions and symptoms. To exploit the potential of serious games in the psychomotor domain, additional variables related to game engagement, such as usefulness, purpose, and user orientation, must be clearly defined and implemented in the context of empirical studies [60].

### This Study

Despite the positive effects of physical activity and the preliminary evidence that serious games have a positive impact on health and physical well-being, new digital technologies [61] should be researched more to become a key measure in the fight against the social- and health care–related challenges, negative effects, and consequences of the pandemic. This intervention study does so by analyzing the effects of the restrictive measures implemented during the COVID 19 pandemic in interaction with exergaming a social and physical activity on seniors' motor skills over the course of a long period. The study is unique insofar as the participants began the intervention before the onset of COVID-19 and carried on during the pandemic (with its restrictive measures). Key questions were, “Can the positive effects reported in the literature be replicated?” and “Can serious games cushion the far-reaching, undoubtedly negative impact of the pandemic measures on the motor skills of seniors?”

## Methods

### MemoreBox

**Figure 1:**
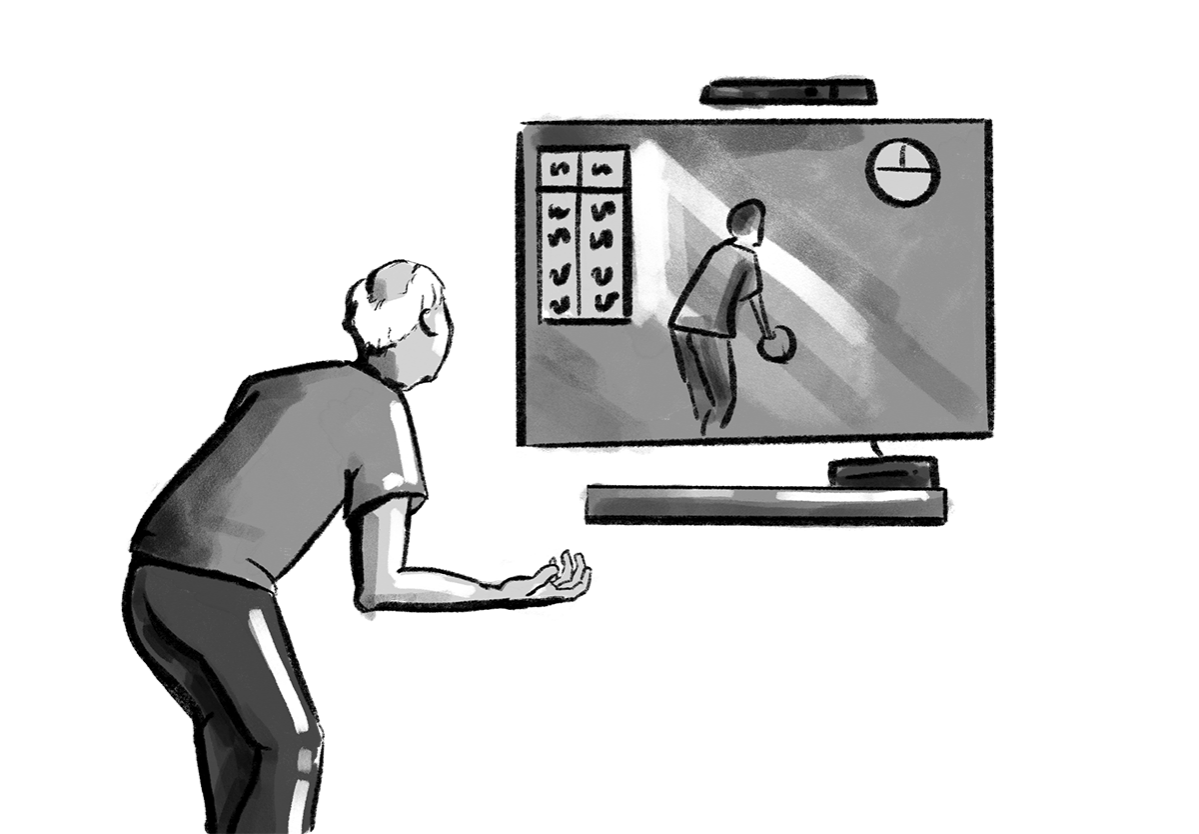
Exemplary Bowling module in the serious game MemoreBox.

In 2014, MemoreBox was developed in response to the need for promoting prevention and health relative to the cognitive, motor, and psychosocial skills of senior citizens and, in particular, nursing home residents. MemoreBox is a health game that includes a gesture-controlled game console, which records the participants' movement data by means of a Kinect camera (Microsoft Corporation) and an individual quick response (QR) code. Games can be played when sitting or standing, individually and in groups, and the therapeutic training program can be used preventively and independently of any indications. There are currently 6 games (motorcycling, bowling [Figure 1], table tennis, singing, postman, and dancing), which are based on everyday activities. Depending on the game, the player’s balance, memory, ability to react, hand-eye coordination, motor skills, stance, or mobility are trained.

### Study Design

As part of a large, applied research study, the data used here for the analysis came from an intervention study that was set up as a quasi-experimental design with repeated measurements. The overarching goal of this intervention study was to test the effectiveness of MemoreBox. To evaluate the game’s effectiveness, we designed a large-scale study with 100 German nursing homes. Over the course of 1 year, we recorded and examined a total of 1000 seniors in an intervention group (IG) and a control group (CG). The intervention, training by playing, was carried out 3 times per week for 1 hour in a group using a fixed training plan that was developed in advance by occupational therapists. Data were collected over the course of 1 year during which participants were asked to complete standardized questionnaires 8 times. The standardized questionnaires on cognition, motor skills, and psychosocial health were administered every 3 months and thus 4-5 times in total. In this paper, we focus on the participants’ motor skills, which were recorded at 4 points over the course of 9 months using the Tinetti test [62].

### COVID-19 Situation

Due to the global COVID-19 pandemic, which has also caused major changes in Germany, at least since March 2020, the research could not be carried out as originally planned. Protection of and care for the target population, that is, vulnerable senior citizens who are dependent on care due to physical or psychological frailties, is the top priority. For a large majority of the participating nursing homes, this situation led to an inevitable interruption of the study and thus of the planned data collection (also due to a lack of appropriate facilities) starting at the beginning of March 2020. However, the situation and the respective restrictions caused by COVID-19 differed greatly from 1 nursing home to the next. Given that such a large number of facilities were originally recruited throughout Germany, a few nursing homes emerged that were able to continue with the study and data collection due to their particular local setups and conditions within the respective facilities. These 11 nursing homes were able to continue the training plan unaltered, which ultimately led to a sample size of 70 participants (IG n=31, 44%, vs CG n=39, 56%).

### Participants

The sample consisted of an IG that played regularly and a CG that did not play. All participants are residents of nursing homes in Germany. We started with 10 participants per nursing home (IG n=5 and CG n=5, 50% each), that is, N=1000. The assignment of groups was voluntary for practical and ethical reasons, which required a final parallelization of the data. Exclusion criteria for both groups were severe mental or neurological illnesses and age below 60 years. In addition, the state of health, comorbidities, and medications of both groups were surveyed.

### Data Collection Process and Instruments

All data collection was carried out by scientifically trained nursing staff. The data collection instruments were designed to suit the target population, and age-related restrictions in the process were considered. This paper deals with the results of the Tinetti test [62], 1 of the standardized questionnaires used to investigate the effectiveness of MemoreBox on the motor performance of seniors in relation to the effects of the pandemic. The Tinetti test is used to evaluate a person's static and dynamic balance skills. The test is divided into 2 components for separate evaluation of gait and balance skills. Both measurement units consist of 8 questions, with the answer options for the walking test being rated from 0 to 2 points and those for the balance test being rated from 0 to 4 points [63]. The maximum total of the Tinetti test is 28 points, made up of 15 points for the balance test and 13 points for the walking test [63].

### Dropouts

**Figure 2:**
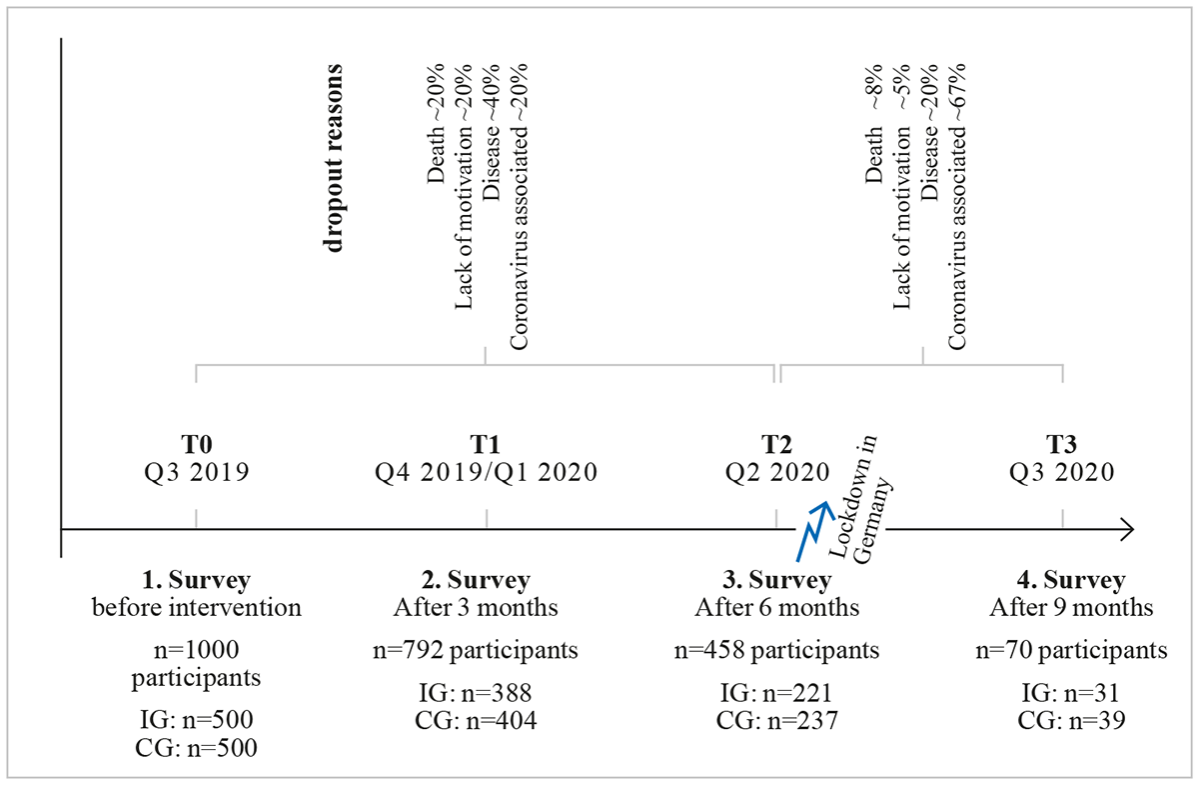
Survey times, periods of operationalization, and dropout reasons. CG: control group; IG: intervention group.

The enormously high dropout rate was largely a result of the outbreak of the COVID-19 pandemic (see description before). Given the length of the study and the target population examined, a high dropout rate was generally assumed. Illnesses and deaths, as well as lack of motivation, were the other main reasons for dropping out. The weightings shown in Figure 2 roughly correspond to those reported in the literature. Over the entire data collection period, the following reasons led to attrition: 409 (44%) seniors dropped out due to COVID-19-associated reasons, 279 (30%) dropped out due to illness, 130 (13%) passed away, and 116 (13%) dropped out due to a lack of motivation.

### Statistical Analysis

At the beginning of the data analysis, the 2 study groups were compared with regard to their characteristics in the dependent variables at baseline (T0). The reason for this was the nonrandomized group assignment, which made *t* tests for independent samples between the groups necessary in order to statistically demonstrate the comparability of the groups. Then, descriptive statistics regarding the groups and relevant sociodemographic variables were carried out. For psychometric calculations, the statistical significance was set at a level of α=.05. To highlight the relevance or practical significance of the results, we calculated the effect size partial η^2^ for mixed-design ANOVA (η^2^<.01, small effect; η^2^<.06, medium effect; η^2^<.14, large effect) [63]. To examine the effects on the IG in comparison to the CG, a 2×3 and a 2×2 mixed-design ANOVA were used in 2 study sections. The dependent variable was motor ability; the factor time, with study segment 1 having 3 levels (T0, T1, T2,) and study segment 2 having 2 levels (T2, T3), acted as an inner-subject factor and group membership as a between-subject factor (2 factor levels). Corresponding Bonferroni corrections within and between subjects were also calculated. The assumptions for the interval scale level of the dependent variable, independence, and the nominal scale level of the between- and within-subject factors can be assumed. Outliers were removed based on the IQR. The assumptions of the normal distribution of the dependent variable residuals, the homogeneity of variance, the equality of the covariances, and the given sphericity can, according to Bortz and Schuster [64], be neglected due to the robustness of the variance analyses if the sample size is almost the same and n>10.

### Ethics Approval

This study was approved by the Ethics Committee of Charite Berlin (Ethikausschuss am Campus Benjamin Franklin; review number: EA4/035/19).

## Results

### Baseline Comparison

Due to the lack of randomization of the study groups, independent sample *t* tests were used for the demographic variables to reveal possible distorting differences between groups. The groups did not differ with regard to the main variables at the beginning of the study (Table 1).

**Table 1:** Mean values, SDs, and statistical differences of the examined variables: IG^a^ and CG^b^ at measurement time T0.

Variables	IG (N=31), mean (SD)	CG (N=39), mean (SD)	Statistics		95% CI	
			*t* (*df*)	*P* value		
Age (years)	85.45 (4.99)	86.66 (8.76)	.718 (60.5)	.48	–0.311 to 0.639	
Level of care needed^c^	2.34 (0.974)	2.47 (0.71)	.592 (61)	.56	–0.347 to 0.645	
State of health^d^	2.33 (1.07)	3.16 (1.22)	–.623 (66)	.54	–0.631 to 0.328	
Health behavior^e^	2.42 (0.720)	2.54 (0.97)	.570 (68)	.57	–0.335 to 0.609	
Health self-assessment^f^	2.84 (0.735)	2.92 (0.81)	.452 (68)	.65	–0.364 to 0.580	
Tinetti mean	1.15 (0.37)	1.05 (0.41)	–.974 (68)	.33	–0.707 to 0.240	
Tinetti total sum	18.16 (5.88)	16.67 (6.58)	–.989 (68)	.33	–0.710 to 0.236	

^a^IG: intervention group (playing regularly).

^b^CG: control group.

^c^0=no need for care to 5=most severe impairment.

^d^0= healthy to 5=chronically ill.

^e^0=does not take care of health to 5=strongly focuses on health.

^f^0=“I rate my health as very bad” to 5=“I rate my health as very good.”

### Descriptive Statistics

The analysis sample was part of the total sample and included those participants who were present at all measurement times and throughout the entire intervention. Therefore, this study included 70 participants. The IG (participants who played regularly) comprised 31 (44%) participants, of which 22 (71%) were female and 9 (29%) male. Participants in the IG ranged were aged from 74 to 97 (mean 85.45, SD 4.99) years. The CG included 31 (79%) females and 8 (21%) males, who were aged from 61 to 102 (mean 86.66, SD 8.76) years. The distribution of age (mean 86.06, SD 8.2 years) and gender (female n=53, 76%, male n=17, 24%) in the total sample roughly corresponded to the findings on the need for care in Germany [65].

### Outcome

Next, the results of the Tinetti data collected from the 70 participants over the course of 1 year were analyzed separately in 2 study sections. Study 1 looked at the first 6 months of the intervention, which analyzed the situation before the outbreak of the pandemic. The second study then dealt with the analysis of the data collected during the pandemic with its severe restrictions for the participants. Both studies looked at the CG in comparison to the IG, which carried out the study plan consistently despite massive, pandemic-related restrictions.

#### Study 1

**Figure 3:**
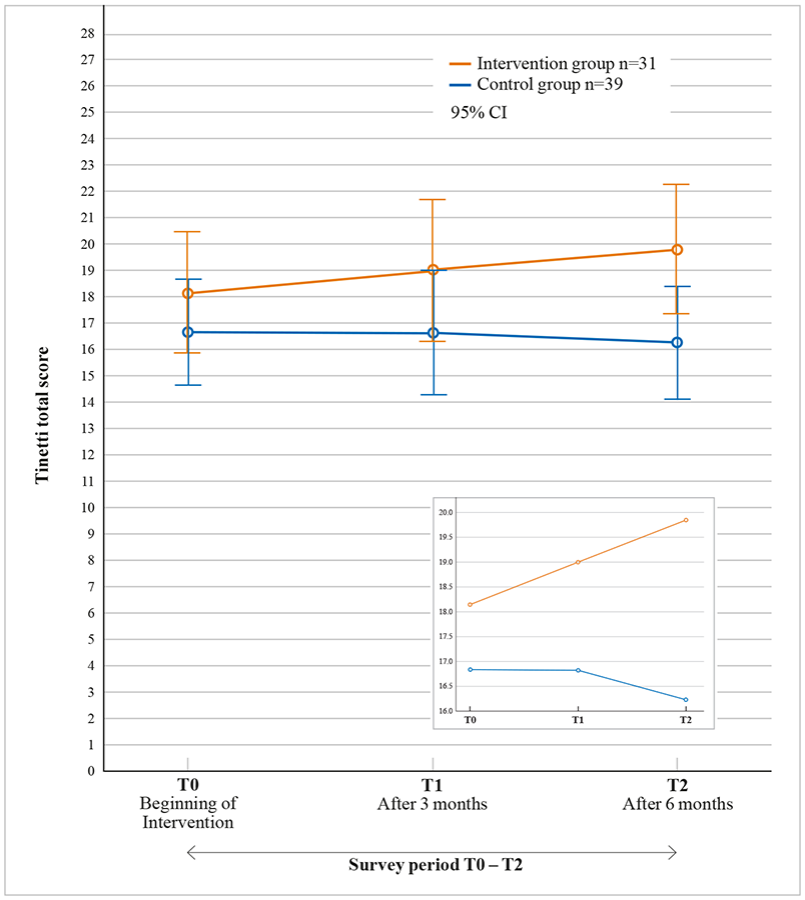
Results of the Tinetti total score for the IG and CG over 6 months of intervention. T0: IG (mean 18.16, SD 5.88), CG (mean 16.67, SD 6.58); T1: IG (mean 19, SD 6.77), CG (mean 16.62, SD 7.93); T2: IG (mean 19.81, SD 6.34), CG (mean 16.23, SD 7.14); CG: control group; IG: intervention group.

Mixed-design ANOVA showed no statistically significant interaction between measurement times and group membership: *F*_2.136_=1.414, *P*<.25, partial η²=.044. Both main effects (time factor: *F*_2.136_=0.489, *P*=.62, partial η²=.016; group membership factor: *F*_1.68_=2.792, *P*=.10, partial η²=.044) showed no statistical significance. However, the post hoc tests carried out within and between subjects indicated a statistically significant difference between the CG (mean 16.23, SD 1.1) and the IG (mean 19.81, SD 1.2) at the third time of measurement (*P*=.02). This finding shows that the regular players differed statistically significantly in their motor skills from the nonplayers after 6 months of playing (Figure 3).

#### Study 2

**Figure 4:**
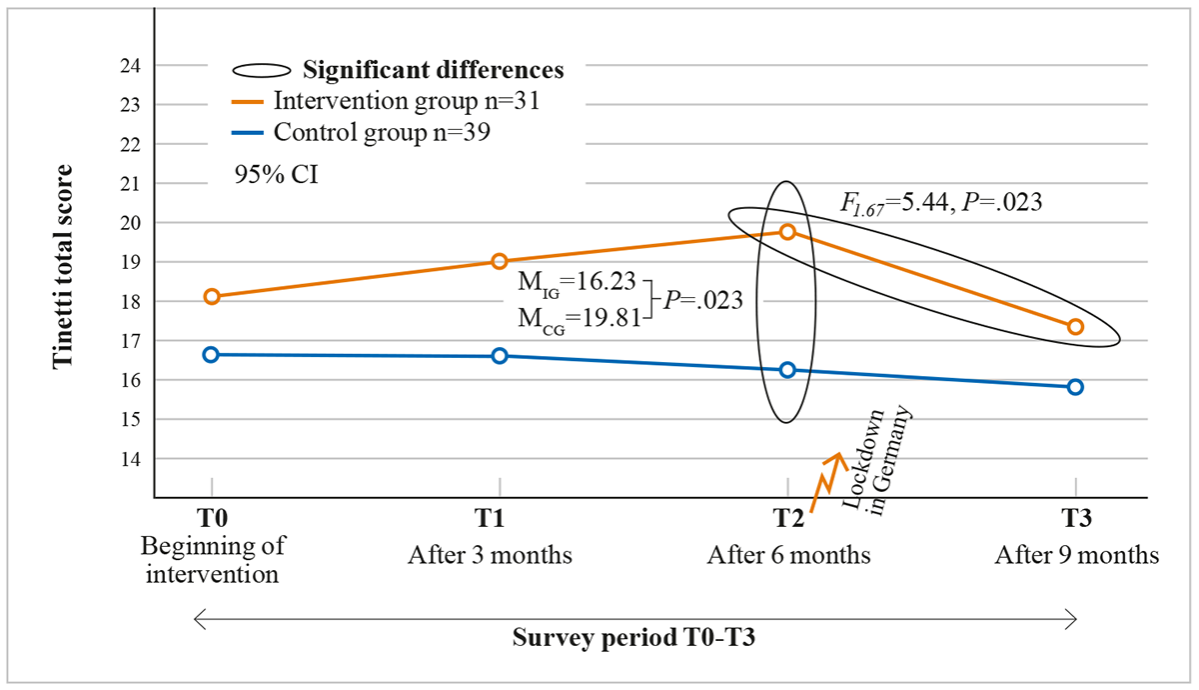
Significant differences after 9 months of intervention between IG and CG and between measurement times. CG: control group; IG: intervention group.

Mixed-design ANOVA used to investigate motor skills before and after the outbreak of the pandemic between the groups did not reveal any statistically significant interaction between measurement times and group membership: *F*_1.67_=2.997, *P*=.09, partial η²=.043. However, there was a statistically significant main effect of the time of measurement, which indicates a significant difference between the measurement times: *F*_1.67_=5.44, *P*=.02, partial η²=.043. However, there was a statistically significant main effect of the time of measurement, which indicates a significant difference between the measurement times: *F*_1.67_=5.44, *P*<.02, partial η²=.075. Considering the descriptive statistics (Figure 4), one can assume a significant drop in estimates between the 2 measurement times. A statistically significant main effect of group membership (*F*_1.67_=2.34, *P*=.12, partial η²=.043) was not found.

## Discussion

### Principal Findings

Senior citizens were particularly affected by the COVID-19 pandemic, which led to far-reaching and extensive measures of isolation. These resulted in a significant reduction in physical activity, which is counterproductive for an independent life and disease prevention. The WHO states that high physical activity is essential for an independent life and disease prevention. Physical inactivity not only leads to deterioration and dependency in physical, psychological, and social areas, it also accelerates the need for nursing and health care services [66].

The goal of this paper was to investigate whether the use of the serious game MemoreBox has a positive impact on the motor skills of seniors in nursing home facilities. Additionally, the data collected provide insights into the effects of COVID-19 restrictions (especially isolating measures) on the development of motor skills of the participants.

### Discussion Study 1

The data analysis of study I clearly shows that during the first 6 months of the study, the IG had increased motor skills, whereas the motor skills of the CG slightly deteriorated. After 6 months, there was a statistically significant difference in motor skills between the 2 groups (Figure 3). These findings are in line with the predominantly promising potential of video game–based interventions (serious games) for the motor skill development of seniors [42-45]. Under known conditions and in the context of a regular everyday life routine, motor stability can be built up, therefore reducing the risk of falling in old age [49]. The results of this study also support the known positive tendencies toward improved cognition in the IG. However, the missing main effects need to be addressed and discussed. Due to the clear trend of the data, it seems conclusive that the time frame for changes in particular is not sufficient. It should also be noted that there are many motor impairments in seniors that cannot be reversed or influenced by training. This might have a major impact on the test results, which do not measure these limitations. It seems imperative to address these points in future research.

### Discussion Study 2

After 6 months of intervention, the outbreak of the COVID-19 pandemic led to severe restrictions in nursing home facilities, which essentially led to isolation and separation, resulting in an overall strong reduction in physical activity. The effects can be clearly seen in the collected data, which show a significant decrease in the motor skills previously gained, resulting in the IG almost falling back to the initial level (Figure 4) and a continued decrease in the CG motor skills as well. These data clearly show the serious effects of isolation measures on motor skills. In line with previous research, the data confirm a deterioration in motor skills, associated with a higher risk of falling [67]. The continued use of serious games does not seem to be able to stop this deterioration in isolation, a finding that may be due to the intensity of the changes. It can be assumed that mobility is negatively affected by the failure of conventional therapeutic, individually tailored, and medically necessary offers such as physical and occupational therapy. Opportunities to exercise without a special therapeutic approach, such as walks outside, excursions, or other therapy-related activities, have also ceased to exist during the pandemic-induced isolation. Isolation reduces social interaction and affects mental health, ultimately leading to reduced physical performance. Regardless of the use of MemoreBox, the lack of social contact in connection with far-reaching changes in daily routines had a considerable negative effect on the overall physical health of the participants.

### Implications

Based on these findings, we conclude that serious games can have a positive influence on the motor skills of seniors. However, despite continued use, other severe motor restrictions (in this case, isolation and separation) can counteract the gain in motor skills. Moreover, regardless of the examined intervention, the data clearly show that COVID-19-related restrictions had a significantly negative influence on the motor skills of the participating seniors. Despite the subsequent limitations, the longitudinal data underlying this study offered a valuable and rare opportunity to examine the direct effects the isolating measures implemented during the COVID-19 pandemic have on senior citizens’ health and mobility. Regarding the reported results on the effectiveness of MemoreBox, this intervention certainly provides a complementary measure to already established prevention programs.

### Limitations

The data collection instrument designed by the study and the examined data have clear limitations. The design limitations, which were analyzed in detail in Kleschnitzki et al [68], are primarily due to the voluntary assignment of the participants to the groups. The lack of randomization is an artifact of the practical implementation.

The most important limitation of this study, however, is the sample size, which implies that the results cannot necessarily be generalized. However, there were at least 30 participants in each study group, which allowed for statistical methods. Furthermore, this population is difficult to reach. This stems from limitations (motor, cognitive, social) with regard to their location. Additionally, the longitudinal design of this study (most studies examine a period of 3 months) also posed great challenges for the participants and led to high dropout rates due to illnesses and deaths. Furthermore, there were large variances within the groups and thus among the participants. This variability, which had a similar range at all measurement times, also posed challenges to making possible significant differences visible. However, the examination of smaller, more homogeneous groups in regard to identifying potential statistically significant differences relative to certain characteristics did not provide any conclusive evidence. The G*Power analysis revealed an a priori minimum sample size of >260 (study 1) and >224 (study 2).

The outbreak of the COVID-19 pandemic undoubtedly had the greatest negative impact on the sample size. It led to high dropout rates among participants and created new challenges for data collection. Nevertheless, it also allowed us to collect data on COVID-19-related changes and associated limitations.

### Future Studies

These limitations and analyzed consequences, in terms of their sustainability, cannot be fully foreseen at the moment and will certainly need to be examined more. The considerable variability in study designs and foci (eg, game applications, data collection instruments, target populations) makes it challenging to compare results from different studies that have examined the effectiveness of serious games [69,70]. Hence, there is a clear need for high-quality, large-scale studies that further investigate the use of serious games by senior citizens. Following a good scientific standard, these studies should focus on the effects that serious games may have on motor skills and fall prevention among seniors. Importantly, studies should consider the limitations of our research and thus include a larger sample size, a longitudinal study design, a randomized trial, and optimized training conditions. It should also be noted that the pandemic provides interesting areas of inquiry for different research domains. The insights gained from pandemic-related research could ultimately help to better understand and counterbalance the negative effects of the COVID-19 pandemic. Finally, the importance and feasibility of using serious games relative to the various issues that the health care system is facing and to the rising number of people in need of care should be investigated further.

### Conclusion

Our results show that digital games can be deployed to promote health in a variety of contexts. They have the potential to break down access barriers and promote social engagement and interaction. The pandemic has highlighted the importance of low-threshold opportunities for exercise and physical activity, especially when conventional recreational programs are either greatly reduced or temporarily not available.

The results of this study help to further establish this new research area by (1) identifying that serious games can have a positive effect on motor skills of senior citizens and (2) revealing critical insights into the effects of COVID-19 on the motor development of seniors in isolation (physical inactivity, increased risk of falling, etc). Additionally, the results are discussed relative to the critical need for action and further research in that area. Lastly, our study contributes to identifying future global health-related challenges as well as potential preventive measures that could be developed and implemented in order to enable seniors, a consistently increasing population, to lead a healthier, independent, and more active lives.

## Abbreviations

CG: control group
IG: intervention group
WHO: World Health Organization
